# Insulin receptor substrates mediate distinct biological responses to insulin-like growth factor receptor activation in breast cancer cells

**DOI:** 10.1038/sj.bjc.6603354

**Published:** 2006-10-17

**Authors:** S A Byron, K B Horwitz, J K Richer, C A Lange, X Zhang, D Yee

**Affiliations:** 1 Department of Pharmacology, University of Minnesota Cancer Center, Minneapolis, MN 55455, USA; 2 Department of Medicine, University of Colorado Health Science Center, Denver, CO 80262, USA; 3 Department of Medicine, University of Minnesota Cancer Center, Minneapolis, MN 55455, USA

**Keywords:** insulin-like growth factor-I, type I insulin-like growth factor receptor, insulin receptor substrate, proliferation, motility

## Abstract

Activation of the type I insulin-like growth factor receptor (IGF-IR) regulates several aspects of the malignant phenotype, including cancer cell proliferation and metastasis. Phosphorylation of adaptor proteins downstream of IGF-IR may couple IGF action to specific cancer phenotypes. In this study, we sought to determine if insulin receptor substrate-1 and -2 (IRS-1 and -2) mediate distinct biological effects in breast cancer cells. Insulin receptor substrate-1 and IRS-2 were expressed in T47D-YA breast cancer cells, which lack IRS-1 and -2 expression, yet retain functional IGF-IR. In the absence of IRS-1 and -2 expression, IGF-IR activation was unable to stimulate proliferation or motility in T47D-YA cells. Expression of IRS-1 resulted in IGF-I-stimulated proliferation, but did not affect motility. In contrast, expression of IRS-2 enhanced IGF-I-stimulated motility, but did not stimulate proliferation. The *α*IR-3, an inhibitor of the IGF-IR, was unable to affect these IGF-stimulated phenotypes unless IRS-1 or -2 was expressed. Thus, IGF-IR alone is unable to regulate important breast cancer cell phenotypes. In these cells, IRS proteins are required for and mediate distinct aspects of IGF-IR-stimulated behaviour. As multiple agents targeting the IGF-IR are currently in early clinical trials, IRS expression should be considered as a potential biomarker for IGF-IR responsiveness.

The evidence implicating the insulin-like growth factor (IGF) system in breast cancer progression suggests that the type I IGF receptor (IGF-IR) may be a viable target for the treatment of breast cancer (Byron and Yee, 2003; Ibrahim and Yee, 2005). Indeed, IGF-IR inhibition, using either dominant negative or pharmacological approaches, has been shown to inhibit breast tumour growth and metastasis both *in vitro* and *in vivo* (Arteaga *et al*, 1989; Arteaga and Osborne, 1989; Burtrum *et al*, 2003; Sachdev *et al*, 2003; Sachdev *et al*, 2004). Given these findings, multiple agents targeting IGF-IR have been developed, with several agents currently in early clinical trials (Garber, 2005). However, as illustrated by the development of the EGFR inhibitor, gefitinib, clinical trial design is crucial towards accurately assessing the effectiveness of a therapeutic approach. In two recent phase II trials in patients with refractory metastatic breast cancer, the EGFR inhibitor gefitinib showed minimal to no clinical activity (Baselga *et al*, 2005; von Minckwitz *et al*, 2005). However, this lack of significant clinical activity was not owing to lack of receptor inhibition in these tumours, but rather to lack of EGFR dependence in the tested population (Baselga *et al*, 2005).

This raises an important question: what molecular attributes will likely be predictive of tumour dependence on IGF-IR? Clearly, IGF-IR expression will be required for response to agents that target the IGF-IR. However, this may not be sufficient to accurately identify breast tumours dependent on IGF-IR. As adaptor molecules are important components of IGF-IR signalling (Zhang and Yee, 2000; Sachdev and Yee, 2001), we propose that expression of the appropriate adaptor molecules, in addition to a functional receptor, may be necessary to observe a therapeutic response to agents targeting IGF-IR.

Although multiple proteins are involved in IGF-IR signal transduction, the insulin receptor substrate (IRS) molecules are the primary family of adaptor proteins used by the IGF-IR. Though six IRS family members have been identified (IRS-1 to IRS-6) (White *et al*, 1985; Tobe *et al*, 1995; Lavan *et al*, 1997a, 1997b; Cai *et al*, 2003), IRS-1 and -2 are the predominant signalling molecules utilised by the IGF-IR to mediate IGF-I action in breast cancer cells (Jackson *et al*, 1998, 2001). Indeed, human breast tumours express both IRS-1 and -2 (Jackson *et al*, 1998; Lee *et al*, 1999).

Previous work from our lab and others suggests that IRS-1 and -2 may mediate different aspects of IGF-IR action in breast cancer cells *in vitro*. Insulin receptor substrate-1 is the predominant signalling molecule activated in MCF-7 breast cancer cells in response to IGF-I stimulation and this activation is required for IGF-I-stimulated cell proliferation of these cells (Jackson *et al*, 1998). In contrast, breast cancer cells selected for metastatic behaviour *in vivo* have increased IRS-2 activation (Jackson *et al*, 2001). In these cells, IGF-IR activation enhanced cell adhesion and motility, suggesting that IRS-2 may mediate these aspects of the malignant phenotype. Thus, IRS-1 activation may correlate with proliferation, whereas IRS-2 may couple to cell migration. These observations may have important clinical implications towards predicting response to anti-IGF-IR strategies. However, these studies merely correlate IGF-IR biology with individual IRS species; studies that directly determine whether IRS-1 and -2 mediate specific aspects of IGF-IR biology in breast cancer cells are needed.

Herein, we sought to determine whether IGF-IR requires IRS-1 and -2 to transduce its biological effects, and whether these IRS proteins mediate distinct aspects of IGF-IR action in breast cancer cells. For these studies, we identified a new cell line to study IRS-dependent effects, the T47D-YA breast cancer cell line. These cells lack IRS-1 and -2 expression, yet express functional IGF-IR, allowing us to investigate whether IGF-IR activation stimulates cell proliferation and motility in the absence of IRS-1 and -2. Furthermore, to determine whether IRS-1 and -2 mediate distinct aspects of IGF-IR biology, we stably expressed IRS-1 or -2 proteins in T47D-YA cells and examined biological responsiveness.

Here, we report that the IGF-IR is unable to mediate biological effects in the absence of IRS-1 and -2 expression, and directly demonstrate that these adaptor molecules mediate distinct aspects of IGF-IR action in breast cancer cells. Furthermore, we propose that expression of IRS adaptor molecules may function as a biomarker for biological responsiveness of the IGF-IR.

## MATERIALS AND METHODS

### Cell culture and reagents

The establishment of the T47D series of breast cancer cell lines occurred in the laboratory of Kathryn Horwitz and has been described previously (Sartorius *et al*, 1994). The T47D-WT, T47D-CO and T47D-Y human breast cancer cells were maintained in Eagle's Minimal Essential Medium supplemented with 5% foetal bovine serum, 6 ng l^−1^ insulin and 10 ml l^−1^ 100X non-essential amino acids. The T47D-YA and T47D-YB cells were maintained in the above medium with 200 *μ*g ml^−1^ of G418. Growth media for T47D-YA IRS-1 and T47D-YA IRS-2 cells was also supplemented with 200 *μ*g ml^−1^ hygromycin. Cells were serum-starved in phenol-red free serum-free media (Improved MEM Zinc Option, supplemented with 20 mm HEPES, 1X Trace Elements, 2 *μ*g ml^−1^ transferrin, and, for IGF-I stimulation, 2 *μ*g ml^−1^ fibronectin). All cells were grown at 37°C in a humidified atmosphere containing 5% CO_2_. The *α*IR-3 (IGF-I receptor Ab-1) was purchased from Calbiochem (San Diego, CA, USA). Insulin-like growth factor-I was purchased from GroPep (Adelaide, Australia) and culture media and fibronectin were purchased from Invitrogen Corporation (Carlsbad, CA, USA).

### Stable transfection

Hemagglutinin-tagged human IRS-1 and -2 cDNAs driven by a CMV promoter in a pcDNA3.1 (–) vector were generously supplied by Adrian Lee (Baylor College of Medicine). Stable IRS-1 and -2 cell clones were generated by co-transfection of T47D-YA cells with IRS-1 or -2 cDNA and pcDNA3.1(–) Hygro^R^ (Invitrogen, Grand Island, NY, USA). Effectene transfection reagent and protocol were followed. Briefly, 8 × 10^5^ cells were plated into 60 mm plates and grown to 50% confluence. Cells were then co-transfected with a 20 : 1 ratio of human IRS-1 or -2 cDNA to plasmid encoding hygromycin resistance and single colonies expanded following antibiotic selection in 200 *μ*g ml^−1^ hygromycin. Positive clones were selected based on positive immunoblot detection of IRS-1 or -2 and the HA tag (data not shown).

### Lysate preparation

Cells were plated in growth medium in a 100 mm dish and grown to 70% confluency. For IGF-I stimulation, cells were washed twice with phosphate-buffered saline (PBS), serum-deprived in serum-free media (SFM) for 24 h and then stimulated with 5 nM IGF-I in SFM for 10 min at 37°C. Cells were washed twice with ice-cold PBS on ice and lysed with 500 *μ*l TNESV lysis buffer (50 mM Tris-Cl (pH 7.4), 1% NP40, 2 mM EDTA (pH 8.0), 100 mM NaCl, 10 mM sodium orthovanadate, 1 mM phenylmethylsulfonyl fluoride, 20 *μ*g ml^−1^ leupeptin and 20 *μ*g ml^−1^ aprotinin). Lysates were cleared by 20 min of centrifugation at 12 000 × *g* at 4°C. Protein concentrations of the lysates were determined using the bicinchoninic acid kit (Pierce, Rockford, IL, USA).

### Immunoblot

Total cell lysates (40 *μ*g) in 1X Laemmli sample buffer were separated using 8% SDS-PAGE and transferred to nitrocellulose. Nitrocellulose membranes were blocked with 5% non-fat dry milk in TBST (0.05% Tween in Tris-buffered saline w/v) for 1 h at room temperature. Primary antibodies were used according to the manufacturer's direction. Insulin receptor substrate-1 (produced by Alpha Diagnostics, San Antonio, TX, USA and protein A agarose affinity purified, as described previously (Sachdev *et al*, 2003)), IRS-2 (Upstate lot #21189, Charlottesville, VA, USA) and IGF-IR (Santa Cruz Biotechnology Inc., Santa Cruz, CA, USA) antibodies were used at 1 : 2000 in 5% milk overnight at 4°C. Total ERK1/2 MAPK (Cell Signaling Technology, Beverly, MA, USA), phosphorylated ERK1/2 MAPK (Thr202/Tyr204) (Cell Signaling Technology, Beverly, MA, USA), and total Akt (Cell Signaling Technology, Beverly, MA, USA) antibodies were used at 1 : 2000 in TBST overnight at 4°C. Phosphorylated Akt (Ser473) (Cell Signaling Technology, Beverly, MA, USA) was used at 1 : 2000 in 1% BSA overnight at 4°C. Following incubation with primary antibody, membranes were washed in TBST six times for 5 min each and incubated for 1 h at room temperature with 1 : 2000 dilution of horseradish peroxide (HRP)-linked anti-rabbit secondary antibody (Amersham, Piscataway, NJ, USA) in 5% milk. Horseradish peroxide (HRP)-conjugated PY-20 antibody (BD Biosciences, San Jose, CA, USA) was used at 1 : 10 000 in TBST for 1 h at room temperature. Membranes were washed six times for 5 min each and chemiluminescence detected using SuperSignal West Pico substrate (Pierce, Rockford, IL, USA). In each experiment, total MAPK levels were determined as a loading control (data not shown).

### Immunoprecipitation

Total cellular lysates (1000 *μ*g) were pre-cleared with 50 *μ*l protein A agarose (Santa Cruz Biotechnology Inc., Santa Cruz, CA, USA). Precleared lysates were incubated overnight at 4°C with IRS-1, IRS-2 or IGF-IR antibody. The next day 50 *μ*l protein A agarose was added for 4 h at 4°C. Immunoprecipitates were collected by centrifugation at 12 000 × *g* for 5 min at 4°C, and washed five times in TNESV buffer. Samples were resuspended in sample buffer, boiled for 5 min, and analysed by SDS-PAGE followed by immunoblotting for phosphorylated tyrosine residues with an HRP-conjugated PY-20 antibody, as described above.

### Monolayer growth assay

Assays were performed as previously described (Twentyman and Luscombe, 1987). Briefly, cells were plated in triplicate in 24-well tissue culture plates at a density of 10 000 cells per well in growth media. After 24 h, cells were washed twice with PBS and switched to SFM for 24 h. Cells were then treated with or without 5 nM IGF-I in SFM and incubated for 6 days. For *α*IR-3 experiments, cells were treated with 3 *μ*g ml^−1^ *α*IR-3, 5 nM IGF-I or 3 *μ*g ml^−1^ *α*IR-3 plus 5 nM IGF-I. Cell number was estimated using the 3-[4,5-dimethyllthiazol 2-yl]2,5-diphenyltetrazolium bromide (MTT) assay. Sixty microlitres of 5 mg ml^−1^ MTT reagent in SFM was added to each well and plates incubated for 3 h at 37°C. Wells were then aspirated and 0.5 ml of solubilising solution (95% DMSO+5% IMEM) added to solubilise the formazan crystals. Absorbance was measured at 570 nm using a 650 nm differential filter.

### Cell cycle analysis

Cells were plated at a density of 0.5 × 10^6^ cells per 60 mm dish. After 24 h, cells were washed twice with PBS and switched to SFM for 48 h. Cells were then treated with or without 5 nM IGF-I in SFM for 24 h, collected on ice in 1 ml of PBS and stained with propidium iodide solution (Krishan, 1975). Cells were analysed for phase of the cell cycle by flow cytometry. Resulting histograms were evaluated using Modfit software.

### Anchorage-independent growth

Anchorage-independent growth assays were performed as previously described (Figueroa *et al*, 1993). Briefly, a bottom agar was prepared by solidifying 1 ml of 0.8% SeaPlaque agarose (BioWhitaker, Rockland, ME, USA) in 1% FBS-containing growth media in each well of a six-well plate. The bottom agar was overlaid with 800 *μ*l of a 0.45% top agar mixture containing 10 000 cells per well in the presence or absence of 5 nM IGF-I treatment and plates incubated at 37°C. After 14 days, colonies were counted using a light microscope with an ocular grid. Only colonies larger than two-thirds of a grid square were counted. Five random fields were counted for each well and fold increase over samples with no IGF-I treatment presented.

### Gold particle assay

Cell motility was measured using a gold particle phagokinetic assay, as previously described (Albrecht-Buehler, 1977; Meyer *et al*, 2001). Briefly, glass coverslips were pre-coated with 1.5 ml of a 5 *μ*g ml^−1^ solution of fibronectin in SFM overnight at 4°C and then coated with a freshly prepared gold particle solution (10.6 mM sodium carbonate, 12.7 mM gold chloride and 0.0087% formaldehyde) and incubated overnight at 4°C to allow the gold particles to precipitate. Cells were grown to 70% confluence, briefly trypsinised and 18 000 cells were plated per coverslip. The cells were allowed to adhere, treatment of SFM with or without IGF-I added, and cells were incubated at 37°C in humidified air with 5% CO_2_ for 24 h. For *α*IR-3 experiments, cells were treated with 3 *μ*g ml^−1^ *α*IR-3, 5 nM IGF-I or 3 *μ*g ml^−1^ *α*IR-3 plus 5 nM IGF-I for 24 h. As the cells move, the gold particles are internalised, leaving a cleared area representing the cell track. Cells were then fixed with 3.5% glutaraldehyde and coverslips mounted on glass slides. Coverslip images were captured using a brightfield microscope with a neutral density filter and the area on the coverslip cleared by cell movement was computed using Simple PCI software.

### Insulin receptor substrate-1 short-interfering RNA

IRS-1 and -2 short-interfering RNA (siRNA) were synthesised by Dharmacon (Lafayette, CO, USA). Four individual sequences were synthesised, and the IRS-1 sequence that best downregulated IRS-1 was selected. Some sequences did not affect IRS-1 levels and these are referred to as non-functional (NF) siRNA. Short-interfering RNA constructs were introduced into MCF-7 cells by lipid transfection using Effectene (QIAGEN, Valencia, CA, USA). To determine the effect of IRS-1 downregulation, we transfected siRNA constructs in triplicate into MCF-7 cells in 96-well plates. After 6 h, the siRNA constructs were removed and cells were further incubated in SFM overnight. Cells were then exposed to 5 nM IGF-I (day 0). Cell number was estimated by MTT assay at day 0 and after an additional 48 h in the presence or absence of 5 nM IGF-I. Results are reported as percent increase over day 0.

### Statistical analysis

Two-way ANOVA with Bonferroni post-test and Student's *t*-test were performed using GraphPad Prism version 3.02 for Windows (GraphPad Software, San Diego, CA, USA).

## RESULTS

### T47D-YA cells lacked insulin-like growth factor-I-stimulated proliferation and motility, despite expression of functional insulin-like growth factor receptor

We utilised a series of T47D breast cancer cell lines that differentially express the two isoforms of the progesterone receptor (PR). The human PR exists in two isoforms, a B isoform (PR-B) and an N-terminal truncated A isoform (PR-A), possessing different transcriptional capacities, despite similar hormone- and DNA-binding properties (Kastner *et al*, 1990; Sartorius *et al*, 1994; Wen *et al*, 1994; Richer *et al*, 2002). T47D cells, here referred to as T47D-WT cells, express both PR-A and -B in an oestrogen-dependent manner (Keydar *et al*, 1979; Sutherland *et al*, 1992). T47D-CO cells express both isoforms of PR independent of ER-*α* function (Horwitz *et al*, 1982). T47D-Y cells express neither PR-A nor PR-B; these cells were transfected with appropriate PR constructs to obtain variants that express only PR-A (T47D-YA) or only PR-B (T47D-YB) (Sartorius *et al*, 1994). These cell lines were of interest to us as PR-B has been shown to regulate IGF-IR signalling by ligand-dependent transcriptional upregulation of IRS-2 (Vassen *et al*, 1999; Cui *et al*, 2003). Evidence also suggests PR-B may regulate IRS-1 expression independent of ligand (unpublished observations). As PR-B may regulate both IRS-1 and -2 levels, we hypothesised that a PR-B-null-cell line may lack IRS-1 and -2 expression. Therefore, we first sought to characterise expression of components of the IGF-IR signalling system in this series of T47D breast cancer cell lines.

**Figure 1:**
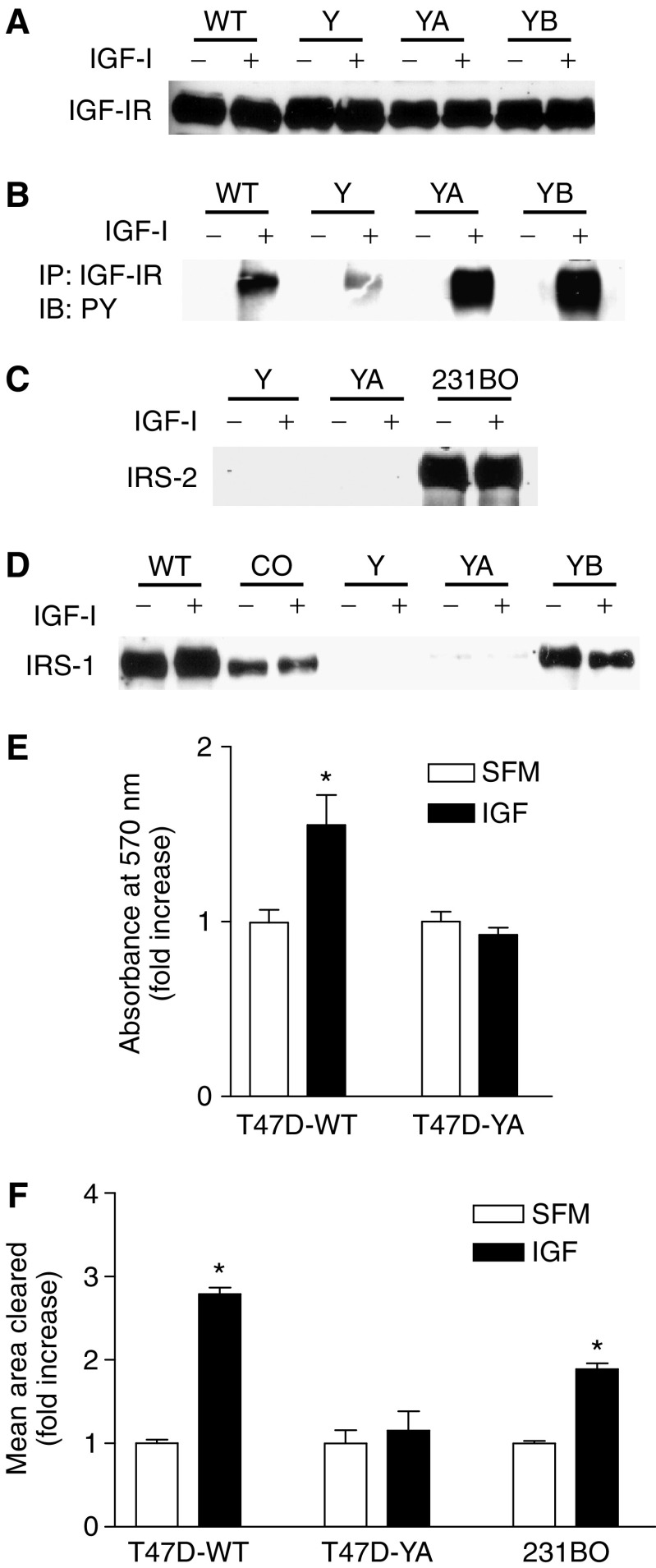
T47D-YA cells lacked IGF-I-stimulated proliferation and motility, despite expression of a functional IGF-IR. (**A**) T47D-WT, T47D-Y, T47D-YA and T47D-YB cells were serum-deprived for 24 h, stimulated with 5 nM IGF-I for 10 min, cell lysates separated by 8% SDS-PAGE and protein levels of the IGF-IR determined by Western blot. (**B**) T47D cell line variants were serum-deprived for 24 h, stimulated for 10 min with 5 nM IGF-I, and cell lysates collected, immunoprecipitated with IGF-IR antibody, and analysed by immunoblotting with an antibody for phosphorylated tyrosine residues. (**C**) T47D-Y and T47D-YA cells were serum-starved for 24 h, stimulated with 5 nM IGF-I for 10 min, cell lysates separated by 8% SDS-PAGE and immunoblotted with anti-IRS-2 antibody. MDA-MB-231BO cells were included as a positive control for IRS-2 expression. (**D**) T47D-WT, T47D-CO, T47D-Y, T47D-YA and T47D-YB cells were stimulated with 5 nM IGF-I for 10 min, cell lysates separated by 8% SDS-PAGE and immunoblotted with anti-IRS-1 antibody. Experiments were performed two to four times and representative data shown. (**E**) T47D-WT and T47D-YA cells were plated in 24-well plates in growth media, switched to SFM after 24 h, treated with or without 5 nM IGF-I for 6 days, and then cell number estimated using an MTT assay. Error bars represent s.e. of the mean and ^*^ represents a significant difference (*P*<0.05) in absorbance in samples treated with IGF-I compared to SFM. *P*-values: T47D-WT (*P*=0.0409) Results shown are representative of three independent experiments. (**F**) T47D-WT and T47D-YA cells were plated on gold particle-coated coverslips. MDA-MB-231BO cells were included as a positive control. The cells were allowed to adhere, treatment of SFM with or without 5 nM IGF-I added, and cells incubated at 37° C for 24 h. Coverslip images were captured using a brightfield microscope with a neutral density filter and the area on the coverslip cleared by cell movement was computed using Simple PCI software. Data are presented as mean area cleared. Error bars represent s.e. of the mean and ^*^ represents a significant difference (*P*<0.05) in samples treated with IGF-I compared to SFM. *P*-value: T47D-WT (*P*<0.001); MDA-MB-231BO (*P*=0.0072). Results shown are representative of three independent experiments.

Each of the T47D cell lines expressed levels of IGF-IR similar to the IGF-I-responsive T47D-WT cell line (Figure 1A). Furthermore, functional IGF-IR was detected in each of the T47D cell lines investigated, with T47D-YA and T47D-YB cells exhibiting levels of activated IGF-IR greater than that of the T47D-WT cells (Figure 1B). Activation of IGF-IR was less in T47D-Y cells than in T47D-WT, T47D-YA or T47D-YB cells. We next investigated expression of IRS-1 and -2 in the T47D cell lines. In the absence of progestins, T47D-Y and T47D-YA cells lacked IRS-2 protein expression (Figure 1C). Furthermore, whereas IRS-1 was expressed in T47D-WT, T47D-CO and T47D-YB cells, neither T47D-Y nor T47D-YA cells expressed detectable levels of IRS-1 protein (Figure 1D). Thus, T47D-Y and T47D-YA cells lacked IRS-1 and -2 expression, yet expressed a functional IGF-IR; T47D-YA cells were selected for additional study owing to their higher level of IGF-I-activated IGF-IR.

To determine whether the IGF-IR could mediate biological responses in the absence of IRS-1 and -2, we next analysed IGF-I-stimulated proliferation and motility in T47D-YA cells. As previously reported, IGF-I stimulated monolayer growth in T47D-WT cells (Figure 1E) (Sutherland *et al*, 1992). However, T47D-YA cells were unresponsive to the mitogenic actions of IGF-I. Furthermore, whereas IGF-I stimulated cell motility of T47D-WT and MDA-MB-231BO cells (positive control) (Jackson *et al*, 2001), IGF-I did not stimulate cell motility in T47D-YA cells (Figure 1F). Together, these data suggest that IGF-IR activation, in the absence of IRS-1 and -2, is insufficient to transduce biological signalling. Furthermore, T47D-YA cells provide an ideal model system to investigate the roles of IRS-1 and -2 in mediating IGF-IR action, as these cells lacked IRS-1 and -2 expression and IGF-I-stimulated proliferation and motility, despite expression of biochemically functional IGF-IR.

### Insulin receptor substrate-1, but not insulin receptor substrate-2, expression resulted in insulin-like growth factor receptor mediated cell proliferation in T47D-YA cells

**Figure 2:**
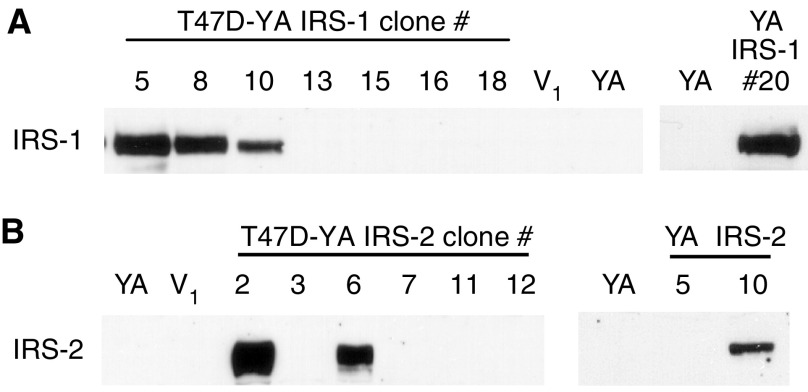
IRS-1 and -2 were stably expressed in T47D-YA cells. (**A**) T47D-YA cells were stably transfected with a cDNA construct encoding HA-tagged human IRS-1. Insulin receptor substrate-1 expression was determined by immunoblot. (**B**) T47D-YA cells were stably transfected with a cDNA construct encoding HA-tagged human IRS-2. Insulin receptor substrate-2 expression was determined by immunoblot.

To determine whether expression of IRS-1 and/or IRS-2 could sufficiently couple IGF-IR to proliferative signalling, T47D-YA breast cancer cells were stably transfected with cDNA constructs encoding human IRS-1 or -2. Insulin receptor substrate-1 expression was detected in T47D-YA/IRS-1 clones #5, #8, #10 and #20 (Figure 2A) and IRS-2 expression detected in T47D-YA/IRS-2 clones #6 and #10 (Figure 2B), which were selected for subsequent studies. T47D-YA/IRS-2 clone #2 was excluded from these studies based on positive expression of both IRS-1 and -2 (data not shown). Similar to T47D-YA cells, vector-transfected control cells had no detectable level of IRS-1 or -2 protein expression.

**Figure 3:**
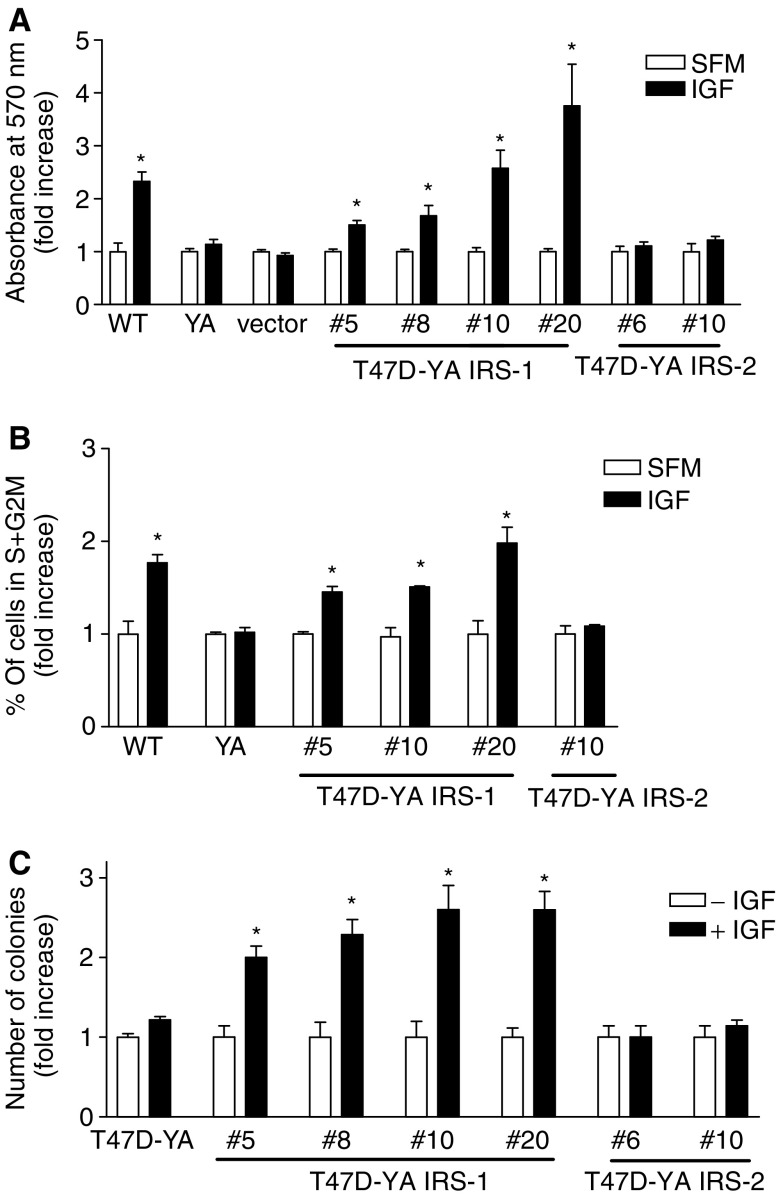
IRS-1, but not IRS-2, expression resulted in IGF-I mediated cell proliferation in T47D-YA cells. (**A**) T47D-YA/IRS-1 cell clones and T47D-YA/IRS-2 cell clones were plated in 24-well plates in serum-containing media, switched to SFM after 24 h, and treated with or without 5 nM IGF-I. After 6 days, cell number was estimated using an MTT assay. Data are represented as fold increase over each cell line's SFM readings. Error bars represent s.e. of the mean and ^*^ represents a significant difference (*P*<0.05) in absorbance in samples treated with IGF-I compared to SFM. *P*-values: T47D-YA/IRS-1 #5 (*P*=0.0013); T47D-YA/IRS-1 #8 (*P*=0.0265); T47D-YA/IRS-1 #10 (*P*=0.0107); T47D-YA/IRS-1 #20 (*P*=0.0247); T47D-WT (*P*=0.0055). Data are representative of three independent experiments. (**B**) T47D-YA/IRS-1 and T47D-YA/IRS-2 cells were plated in 60 mm dishes in serum-containing media and, after 24 h, switched to SFM for 48 h. Cells were then treated with or without 5 nM IGF-I in SFM for 24 h, stained with propidium iodide, and analysed for phase of the cell cycle by flow cytometry. Error bars represent s.e. of the mean and ^*^ represents a significant difference (*P*<0.05) in % of cells in S+G_2_M phases of the cell cycle in samples treated with IGF-I compared to SFM. *P*-values: T47D-WT (*P*=0.045), T47D-YA/IRS-1 #5 (*P*=0.020), T47D-YA/IRS-1 #10 (*P*=0.0317), T47D-YA/IRS-1 #20 (*P*=0.0483). (**C**) A bottom agar was prepared and overlaid with 800 *μ*l of a 0.45% top agar mixture containing 10 000 cells per well in the presence or absence of 5 nM IGF-I treatment and plates incubated at 37°C. After 14 days, colonies formed in the soft agar assay were counted using a light microscope with an ocular grid. Only colonies larger than two-thirds of a grid square were counted. Five random fields were counted for each well and the average number of colonies per well calculated. Results are presented as fold increase over each cell line's SFM values. Results are representative of three experiments performed in triplicate for each treatment. Error bars represent s.e. of the mean and ^*^ represents a significant difference (*P*<0.05) in samples treated with IGF-I compared to SFM. *P*-values: T47D-YA/IRS-1 #5 (*P*=0.0078), T47D-YA/IRS-1 #8 (*P*=0.0086), T47D-YA/IRS-1 #10 (*P*=0.0119), T47D-YA/IRS-1 #20 (*P*=0.0034).

We next investigated whether introduction of IRS-1 or -2 expression in T47D-YA cells was sufficient to couple the IGF-IR to proliferation. As shown in Figure 3A, the T47D-YA/IRS-1 cells exhibited an increase in monolayer growth upon IGF-I treatment, similar to T47D-WT cells, demonstrating that introduction of IRS-1 expression was sufficient to induce IGF-IR-mediated stimulation of monolayer growth in T47D-YA cells. In contrast, introduction of IRS-2 expression did not stimulate IGF-IR-mediated monolayer growth in T47D-YA cells. Similar to T47D-YA cells, vector-transfected cells did not exhibit IGF-I-stimulated monolayer growth.

To further demonstrate that IRS-1 couples the IGF-IR to proliferative pathways, we measured IGF-I-stimulated cell cycle progression by flow cytometry. As shown in Figure 3B, IGF-I was unable to stimulate T47D-YA cells to enter S phase of the cell cycle. However, T47D-YA/IRS-1 cells exhibited IGF-I-stimulated entry into S phase, similar to T47D-WT cells. In contrast, IGF-I did not stimulate cell cycle progression in T47D-YA/IRS-2 cells. All cell lines were growth responsive to medium containing 5% foetal bovine serum (data not shown). To further investigate IGF-IR-mediated proliferation of T47D-YA/IRS-1 cells, we assessed anchorage-independent growth using a soft agar assay. As shown in Figure 3C, T47D-YA/IRS-1 cells exhibited an IGF-I-stimulated increase in colony formation, compared to T47D-YA cells. In contrast, T47D-YA/IRS-2 cells exhibited soft agar growth characteristics similar to T47D-YA cells. Together, these results suggest that IRS-1 specifically mediates the proliferation signals of the IGF-IR and that expression of IRS-1 is sufficient to establish IGF-IR-mediated monolayer and anchorage-independent growth in T47D-YA cells.

### Expression of insulin receptor substrate-2, but not insulin receptor substrate-1, resulted in insulin-like growth factor receptor-stimulated motility in T47D-YA cells

**Figure 4:**
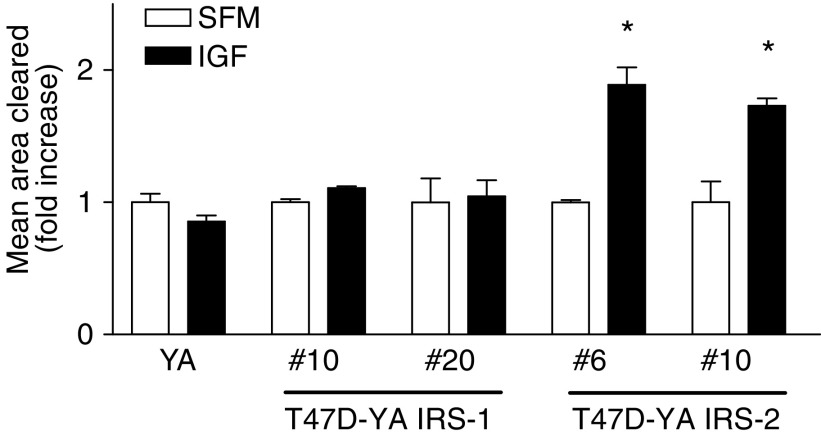
Expression of IRS-2, but not IRS-1, resulted in IGF-I-stimulated motility in T47D-YA cells. T47D-YA/IRS-1 and T47D-YA/IRS-2 cells were plated on gold particle-coated coverslips. The cells were allowed to adhere, treatment of SFM with or without 5 nM IGF-I added, and cells incubated at 37°C for 24 h. Coverslip images were captured using a brightfield microscope with a neutral density filter and the area on the coverslip cleared by cell movement was computed using Simple PCI software. Data are presented as mean area cleared. Error bars represent s.e. of the mean and ^*^ represents a significant difference (*P*<0.05) in samples treated with IGF-I compared to SFM. *P*-values: YA/IRS-2 #6 (*P*=0.0224); YA/IRS-2 #10 (*P*=0.0481); Results are representative of three independent experiments.

Previous work by our lab and others has implicated a role for IRS-2 in mediating IGF-IR-stimulated cell motility. To investigate whether IRS-2 specifically mediates this biological action of the IGF-IR, we measured IGF-I-stimulated motility of T47D-YA cells expressing IRS-1 or -2. Although Boyden chambers have been used to evaluate motility, in this study we used the gold particle assay to avoid the confounding effects of IGF signalling on cell adhesion (Zhang *et al*, 2004). Whereas IGF-IR activation did not stimulate cell motility in T47D-YA cells, IGF-I was able to stimulate cell motility in T47D-YA/IRS-2 cells (Figure 4). In contrast, IGF-I did not stimulate cell motility in T47D-YA/IRS-1 cells, suggesting a functional specificity of IRS-2 in IGF-IR-mediated cell motility.

### Downregulation of insulin receptor substrate-1 diminished response to insulin-like growth factor-I in MCF-7 cells

**Figure 5:**
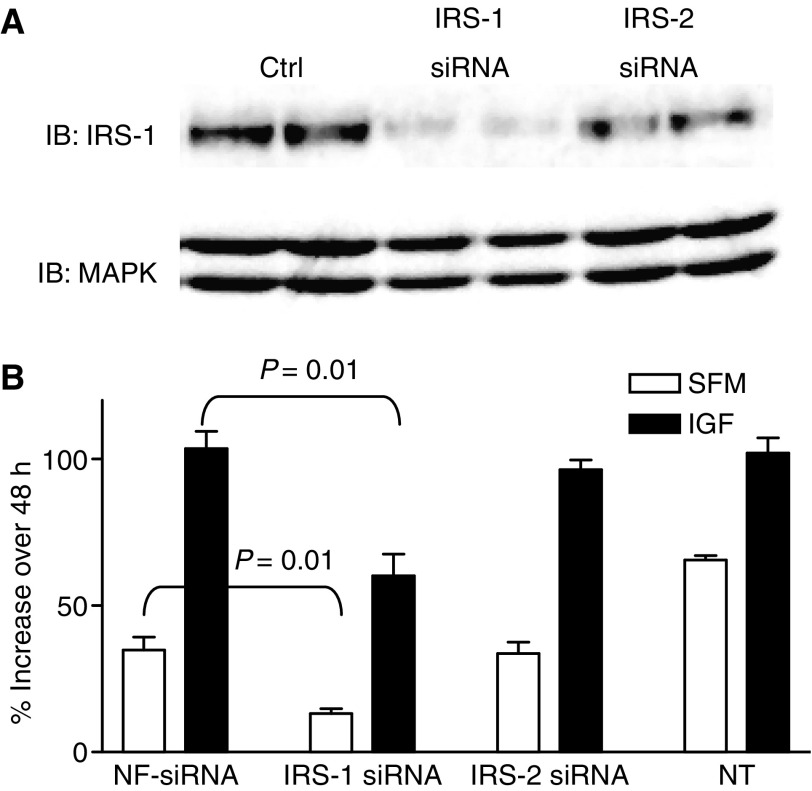
Downregulation of IRS-1 inhibited response to IGF-I in MCF-7 cells. (**A**) MCF-7 breast cancer cells were transfected with IRS-1, IRS-2 or nonfunctional (NF) control siRNA constructs. After 24 h, cells were lysed and examined for IRS-1 expression by immunoblotting. Total MAPK was used as a loading control. (**B**) Effect of IRS-1 downregulation on IGF-I-stimulated growth was examined in 96-well plates. Cells were transfected with a NF, IRS-1 or IRS-2 siRNA for 6 h. Non-transfected (NT) cells were also examined. After incubation in SFM overnight, cells were treated in the absence (open bar) or presence (black bar) of 5 nM IGF-I. Cell number was estimated at this time point (day 0) by MTT assay. After 48 h, cell numbers were again determined and results are presented as percent increase over day 0. These treatments were carried out in triplicate and results were repeated. A representative experiment is shown. Statistically significant differences are shown.

We have previously shown that downregulation of IRS-2 in MDA-MB-231BO cells inhibited IGF-stimulated motility (Jackson *et al*, 2001). However, it has been more difficult to examine the function of IRS-1 in breast cancer cells as stable transfection of an antisense IRS-1 expression vector is not tolerated by breast cancer cells (data not shown). To address this problem, we transiently transfected IRS-1 siRNA into MCF-7 cells. As shown in Figure 5A, IRS-1 siRNA downregulated IRS-1 whereas an IRS-2 construct did not. Signalling downstream of IRS-1 was inhibited proportionally to the level of IRS-1 downregulation (data not shown). Inhibition of IRS-1 diminished IGF-I-stimulated growth (Figure 5B). In contrast, a NF and IRS-2 siRNA had no effect on IGF-I stimulation. It is noteworthy that downregulation of IRS-1 also inhibited the basal growth of MCF-7 cell suggesting an important role for this protein even in the absence of IGF-signalling. Taken with our previously published reports, these data support a role for IRS-1, but not IRS-2, in IGF-stimulated growth of breast cancer cells.

### Insulin receptor substrate-1 and -2 selectively coupled the insulin-like growth factor receptor to downstream pathways

**Figure 6:**
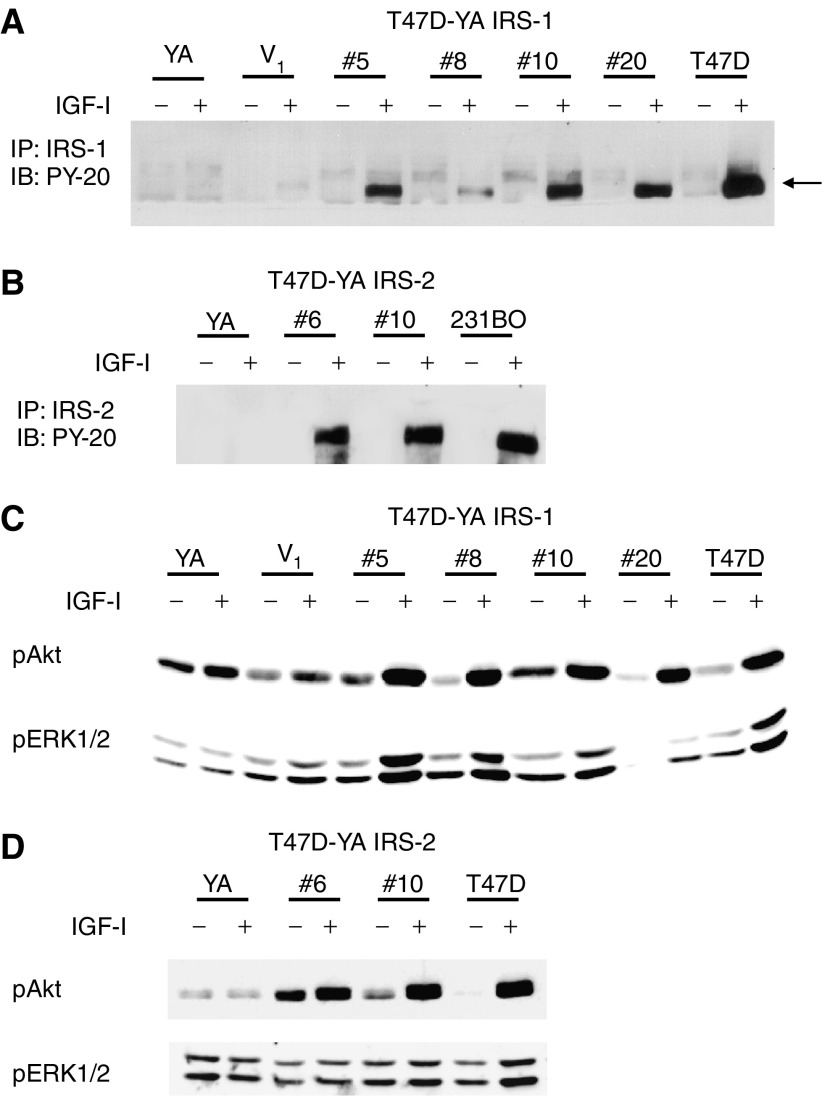
IRS-1 and -2 selectively coupled the IGF-IR to downstream pathways. (**A**) T47D-YA/IRS-1 cells were stimulated with 5 nM IGF-I for 10 min and cell lysates analysed for activated IRS-1 by immunoprecipitating IRS-1, followed by immunoblotting for phosphorylated tyrosine residues. (**B**) T47D-YA/IRS-2 cells were stimulated with 5 nM IGF-I for 10 min and cell lysates analysed for activated IRS-2 by immunoprecipitating IRS-2, followed by immunoblotting for phosphorylated tyrosine residues. (**C**) T47D-YA/IRS-1 cells were examined by immunoblot for MAPK and PI3K activation using phosphorylation specific antibodies following treatment with 5 nM IGF-I for 10 min. (**D**) T47D-YA/IRS-2 cell clones were examined by immunoblot for MAPK and PI3K activation after treatment with 5 nM IGF-I for 10 min. Experiments were repeated a minimum of three times, and representative blots shown.

We next sought to investigate the mechanistic basis for the specific biological actions of IRS-1 and -2. To verify that both IRS-1 and -2 were functionally competent to participate in IGF-IR signalling, tyrosine phosphorylated IRS-1 or -2 was detected by immunoprecipitation and anti-phosphotyrosine immunoblotting. As shown in Figure 6A, each of the T47D-YA/IRS-1 cell clones exhibited IGF-I-stimulated tyrosine phosphorylation of IRS-1. In addition, IRS-2 was phosphorylated upon IGF-I stimulation in each of the IRS-2-transfected cell clones (Figure 6B). Insulin-like growth factor-I treatment did not result in tyrosine phosphorylation of IRS-1 or -2 in T47D-YA cells, as was expected owing to the lack of IRS-1 and -2 expression in these cells. Thus, transfected IRS-1 and -2 were both able to participate in IGF-IR signalling.

To determine whether IRS-1 and -2 couple IGF-IR to distinct downstream pathways, we used phosphorylation-specific antibodies to measure activation of PI3K and MAPK pathways, two of the predominant signalling pathways activated by IGF-IR. In T47D-YA/IRS-1 cells, IGF-I stimulated phosphorylation of both Akt (a downstream target of PI3K activation) and MAPK (Figure 6C). However, in T47D-YA/IRS-2 cells, IGF-I stimulated phosphorylation of Akt, but not MAPK (Figure 6D). Together, these data suggest that, whereas both IRS-1 and -2 couple IGF-IR to PI3K activation in T47D-YA cells, IRS-1 may selectively couple IGF-IR to MAPK activation.

### Insulin receptor substrate-1 and -2 were required for sensitivity to anti-insulin-like growth factor receptor strategies

**Figure 7:**
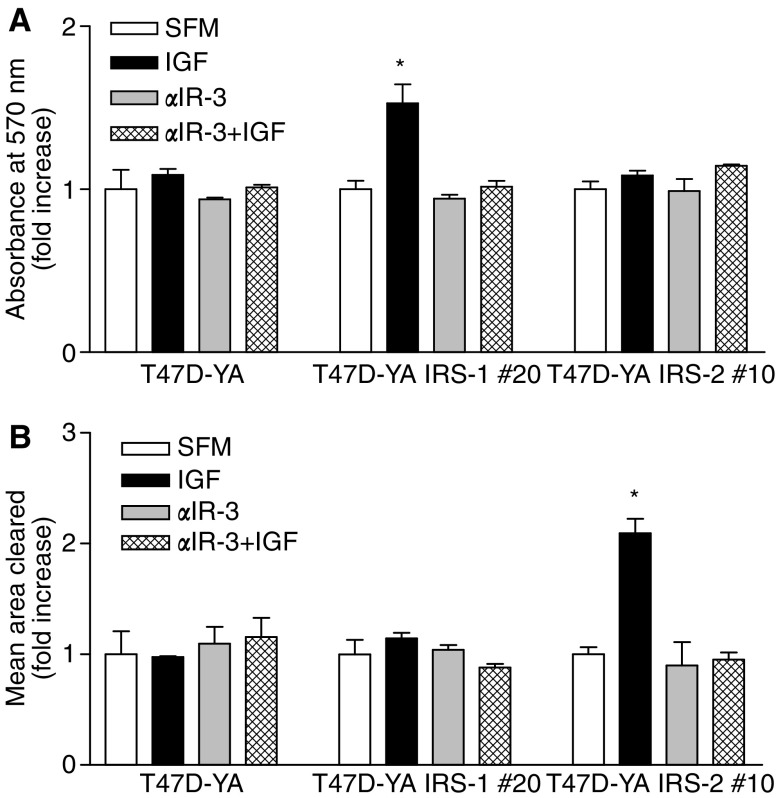
IRS-1 and -2 were required for sensitivity to anti-IGF-IR strategies. (**A**) T47D-YA, T47D-YA/IRS-1, and T47D-YA/IRS-2 cells were plated in triplicate in 24-well tissue culture plates at a density of 10 000 cells per well in growth media. After 24 h, cells were washed twice with 1 × PBS and switched to SFM for 24 h. Cells were then treated with SFM, 3 *μ*g ml^−1^ *α*IR-3, 5 nM IGF-I or 3 *μ*g ml^−1^ *α*IR-3 plus 5 nM IGF-I for 5 days and cell number estimated using the MTT assay. Data are represented as fold increase over each cell line's SFM readings. Error bars represent s.e. of the mean and ^*^ represents a significant difference (*P*<0.05) in absorbance in samples treated with IGF-I compared to SFM. *P*-values: T47D-YA/IRS-1 #20 (*P*=0.0141). Data are representative of three independent experiments. (**B**) T47D-YA, T47D-YA/IRS-1 and T47D-YA/IRS-2 cells were plated on gold particle-coated coverslips. Cells were allowed to adhere and then treated with SFM, 3 *μ*g ml^−1^ *α*IR-3, 5 nM IGF-I or 3 *μ*g ml^−1^ *α*IR-3 plus 5 nM IGF-I for 24 h. Coverslip images were captured using a brightfield microscope with a neutral density filter and the area on the coverslip cleared by cell movement was computed using Simple PCI software. Data are presented as fold increase of the mean area cleared compared to each cell line's SFM readings. Error bars represent s.e. of the mean and ^*^ represents a significant difference (*P*<0.05) in samples treated with IGF-I compared to SFM. *P*-values: YA/IRS-2 #10 (*P*=0.0169). Results are representative of three independent experiments.

Given the current development of anti-IGF-IR strategies in clinical trials, we next sought to determine whether expression of IRS proteins was required for cell sensitivity to IGF-IR inhibition. To address this question, we utilised *α*IR-3, a monoclonal antibody targeted against IGF-IR that has previously been shown to disrupt IGF-IR signalling and inhibit growth of some breast cancer cells both *in vitro* and *in vivo* (Arteaga, 1992). As shown in Figure 7A, *α*IR-3 inhibited IGF-I stimulation of monolayer growth in T47D-YA/IRS-1 cells, but did not affect the monolayer growth of T47D-YA or T47D-YA/IRS-2 cells. Furthermore, *α*IR-3 treatment also inhibited IGF-I-stimulated cell motility in the T47D-YA/IRS-2 cells, yet did not inhibit cell motility in T47D-YA or T47D-YA/IRS-1 cells (Figure 7B). Together, these data suggest that the biological response to IGF-I stimulation, as well as sensitivity to an anti-IGF-IR agent, is dependent not only on expression of functional IGF-IR, but also on expression of the appropriate adaptor molecule.

## DISCUSSION

Our previous data suggested that the IRS proteins were the predominant molecules phosphorylated by IGF-IR in breast cancer cells. Here, we show that IGF-IR requires these adaptor molecules to mediate its biological effects, as exemplified by the lack of IGF-IR stimulated proliferation or motility in T47D-YA cells, which lack IRS-1 and -2 expression, yet express biochemically functional IGF-IR. Among cancer cell lines, the T47D-YA cells are unique. Variants of MCF-7 cells selected for loss of oestrogen receptor lose both IGF-IR and IRS-1 expression, the T47D variants lose only the adaptor proteins (Oesterreich *et al*, 2001), Thus, they represent an ideal system to study the specific function of IGF-IR with and without expression of the specific adaptor proteins. Our data provide direct evidence that IRS-1 and -2 couple the IGF-IR to distinct phenotypes, with IRS-1 specifically mediating IGF-IR-stimulated proliferation, and IRS-2 mediating the motility actions of the IGF-IR.

Our results demonstrate that stable expression of IRS-1 in T47D-YA cells restores IGF-IR signalling through IRS-1 and sensitises these cells to IGF-I-stimulated proliferation. In contrast to T47D-YA/IRS-1 cells, T47D-YA/IRS-2 cells did not proliferate in response to IGF-I. In breast cancer cells, IRS-1 overexpression has been associated with tumour development and hormone independence (Surmacz, 2000). In addition, high levels of IRS-1 expression have been shown to correlate with early disease recurrence in oestrogen receptor-positive primary breast tumours (Rocha *et al*, 1997; Lee *et al*, 1999). Importantly, our results suggest that growth regulation of breast cancer by the IGF-IR is specifically dependent on IRS-1-mediated signalling. Downregulation of IRS-1 is not well tolerated by breast cancer cells. However, our data using transient transfection in MCF-7 cells suggest that IRS-1 is the key regulator of IGF-IR-stimulated proliferation.

Our data also support a specific functional role for IRS-2 in IGF-IR-stimulated cell motility. Previous work in our lab suggests a role for IRS-2 in mediating cell motility in highly metastatic breast cancer cell lines (Jackson *et al*, 2001). Furthermore, elegant studies by Nagle *et al* (2004) recently used a mouse model of mammary tumorigenesis and IRS-2-null mice to conclude that IRS-2 is an important mediator of mammary tumour metastasis. Here, expression of IRS-2 in T47D-YA cells coupled IGF-IR to stimulation of cell motility, whereas IRS-1 expression did not enhance the motility response of T47D-YA cells to IGF-I. Despite the consistency of these data, reports from other labs have suggested a role for IRS-1 in increasing cell adhesion and decreasing cell motility in prostate cancer cells (Reiss *et al*, 2001). This may be a cell type specific effect or require interplay with pathways not expressed in T47D breast cancer cells. Furthermore, cell migration is a highly complex process (Shaw, 2001; Zhang *et al*, 2004). In our model system, we used fibronectin to engage specific integrin receptors. It is possible that motility mediated through other pathways, such as IRS-1, could be dependent on integrin attachment to other extracellular matrix substrates.

The specificity of IRS-1 and -2 to mediate IGF-IR-stimulated proliferation and motility, respectively, may be a result of coupling the IGF-IR to distinct downstream signalling pathways. Data presented here suggest that, whereas both IRS-1 and -2 couple to PI3K activation, IGF-IR stimulates MAPK signalling only in T47D-YA cells expressing IRS-1. Indeed, a direct correlation between IRS-1 tyrosine phosphorylation levels, activation of PI3K and MAPK, and cell proliferation has been observed (Lee *et al*, 1999). Furthermore, chemical inhibition of PI3K and MAPK (using LY294002 and UO126, respectively) was sufficient to inhibit IGF-I-stimulated entry into S phase in T47D-YA/IRS-1 cells (data not shown), suggesting that activation of both MAPK and PI3K pathways is required for IRS-1 to link IGF-IR to IGF-I-stimulated proliferation.

However, we have also shown that IRS-1 expression alone is insufficient to couple IGF-IR signalling to proliferative pathways in all cells. We have previously transfected the MDA-MB-468 and MDA-MB-435 cell lines with IRS-1 (Jackson and Yee, 1999). MDA-MB-468 cells have been shown to be deficient in IRS-1 expression (Sepp-Lorenzino *et al*, 1994). MDA-MB-435 cells are unable to proliferate in response to IGF-I (Sachdev *et al*, 2004), yet have IGF-stimulated motility. Both of these cell lines have constitutively active MAPK. Thus, the ability of IRS-1 to stimulate proliferation may lie in the ability of this adaptor protein to couple IGF-IR activation to MAPK. If cells already have other pathways that activate MAPK, then the role of IGF-IR/IRS-1 is likely superfluous. Our studies also suggest it may be difficult to determine the function of IRS proteins in cells merely by introducing the protein into IRS-deficient cell lines. Although this approach can successfully identify signalling pathways initiated by IRS proteins (Wang *et al*, 1993; Sun *et al*, 1997; Valentinis *et al*, 2000), our data show it may be difficult to discern the biology regulated by these activated pathways using cells that never expressed these proteins.

Insulin-like growth factor-I treatment did not stimulate MAPK signalling in T47D-YA/IRS-2 cells, suggesting that IGF-IR stimulation of cell motility does not require stimulation of MAPK signalling. Recent work from our lab and others suggests that activation of multiple signalling pathways is required for IGF-IR stimulation of cell motility (Zhang *et al*, 2005). In the highly metastatic MDA-231BO cell line, IGF-IR stimulation of cell motility is dependent on IRS-2 expression, PI3K and p38 activation and integrin ligation (Jackson *et al*, 2001; Zhang and Yee, 2002; Zhang *et al*, 2004; Zhang *et al*, 2005). However, we were unable to show p38 activation in T47D-YA/IRS-2 cells (data not shown), suggesting that the mechanism of IGF-IR-mediated motility is cell-type dependent. Thus, the capacity of IRS-2 to mediate IGF-IR-stimulated cell motility is likely dependent on selective coupling to activation of additional required downstream signalling pathways that remain to be determined.

These findings have important implications, as several agents targeting IGF-IR are currently in Phase I clinical trials. In breast cancer, the value of measuring relevant biomarkers in the conduct of clinical trials has been well documented. As modelled by Pegram *et al* (2005) patient selection for the testing of novel cancer therapeutics is critical. As agents targeting the IGF-IR prepare to enter Phase II clinical trials, we must thoughtfully consider how to best select patients with tumours dependent on IGF-IR. Our data presented here suggest that expression of biochemically functional IGF-IR alone may not couple to clinically measurable phenotypes. This is well illustrated by the T47D-YA breast cancer cells, which express and activate IGF-IR, but fail to translate this activation into stimulation of biologically relevant responses. Indeed, we demonstrate here that *α*IR-3, an inhibitor of IGF-IR, did not inhibit monolayer growth or cell motility of T47D-YA cells. However, introduction of IRS-1 or -2 expression in T47D-YA cells was sufficient to introduce sensitivity to *α*IR-3, suggesting that expression of appropriate adaptor molecules, in addition to functional receptor, may be necessary to observe a response to therapeutic agents targeting IGF-IR.

The success of anticancer agents in phase II clinical trials is typically measured by objective tumour response rate, and does not take into account alterations in other tumour phenotypes, such as cancer metastasis. Our data show that expression of specific adaptor proteins downstream of IGF-IR regulates distinct phenotypes. Although both IRS-1 and -2 regulate the biological effects of IGF-IR, only inhibition of proliferation is commonly measured in phase II clinical trials. Thus, we propose that IRS-1 expression, by linking IGF-IR to downstream signalling pathways required for proliferation, may serve as a functional biomarker of IGF-IR activity. At a minimum, measurement of key adaptor proteins (IRS-1, IRS-2, phospho MAPK and phospho Akt), in addition to measurement of receptor expression, should be considered when conducting clinical trials of IGF-IR antagonists. Detection of these proteins in formalin-fixed tissue would be ideal and validation of reagents to detect these proteins must be carefully performed.

In summary, we provide evidence that, though a functional IGF-IR is a necessary mediator of IGF-I action, biological signalling of the IGF-IR requires expression of appropriate adaptor molecules suggesting that determination of IGF-IR expression is insufficient to predict IGF-IR dependency of a tumour. Furthermore, the cellular response following IGF-IR stimulation is dependent on the species of IRS utilised by the IGF-IR, with IRS-1 mediating IGF-IR-stimulated proliferation and IRS-2 mediating IGF-IR-stimulated cell motility. Thus, the requirement of IRS expression for IGF-IR action reported here should be considered to effectively design Phase II clinical trials for agents targeting the IGF-IR. By coupling IGF-IR activation to proliferative pathways, expression of IRS-1 could identify tumours that are most likely to respond to IGF-IR inhibition.

## References

[bib1] Albrecht-Buehler G (1977) The phagokinetic tracks of 3T3 cells. Cell 11: 395–404 329998 10.1016/0092-8674(77)90057-5

[bib2] Arteaga CL (1992) Interference of the IGF system as a strategy to inhibit breast cancer growth. Breast Cancer Res Treat 22: 101–106 1421419 10.1007/BF01833338

[bib3] Arteaga CL, Kitten LJ, Coronado EB, Jacobs S, Kull Jr FC, Allred DC, Osborne CK (1989) Blockade of the type I somatomedin receptor inhibits growth of human breast cancer cells in athymic mice. J Clin Invest 84: 1418–1423 2553774 10.1172/JCI114315 PMC304004

[bib4] Arteaga CL, Osborne CK (1989) Growth inhibition of human breast cancer cells *in vitro* with an antibody against the type I somatomedin receptor. Cancer Res 49: 6237–6241 2553250

[bib5] Baselga J, Albanell J, Ruiz A, Lluch A, Gascon P, Guillem V, Gonzalez S, Sauleda S, Marimon I, Tabernero JM, Koehler MT, Rojo F (2005) Phase II and tumor pharmacodynamic study of gefitinib in patients with advanced breast cancer. J Clin Oncol 23: 5323–5333 15939921 10.1200/JCO.2005.08.326

[bib6] Burtrum D, Zhu Z, Lu D, Anderson DM, Prewett M, Pereira DS, Bassi R, Abdullah R, Hooper AT, Koo H, Jimenez X, Johnson D, Apblett R, Kussie P, Bohlen P, Witte L, Hicklin DJ, Ludwig DL (2003) A fully human monoclonal antibody to the insulin-like growth factor I receptor blocks ligand-dependent signaling and inhibits human tumor growth *in vivo*. Cancer Res 63: 8912–8921 14695208

[bib7] Byron SA, Yee D (2003) Potential therapeutic strategies to interrupt insulin-like growth factor signaling in breast cancer. Semin Oncol 30: 125–132 14613033 10.1053/j.seminoncol.2003.08.014

[bib8] Cai D, Dhe-Paganon S, Melendez PA, Lee J, Shoelson SE (2003) Two new substrates in insulin signaling, IRS5/DOK4 and IRS6/DOK5. J Biol Chem 278: 25323–25330 12730241 10.1074/jbc.M212430200

[bib9] Cui X, Lazard Z, Zhang P, Hopp TA, Lee AV (2003) Progesterone crosstalks with insulin-like growth factor signaling in breast cancer cells via induction of insulin receptor substrate-2. Oncogene 22: 6937–6941 14534541 10.1038/sj.onc.1206803

[bib10] Figueroa JA, Sharma J, Jackson JG, McDermott MJ, Hilsenbeck SG, Yee D (1993) Recombinant insulin-like growth factor binding protein-1 inhibits IGF-I, serum, and estrogen-dependent growth of MCF-7 human breast cancer cells. J Cell Physiol 157: 229–236 7693722 10.1002/jcp.1041570204

[bib11] Garber K (2005) IGF-1: old growth factor shines as new drug target. J Natl Cancer Inst 97: 790–792 15928295 10.1093/jnci/97.11.790

[bib12] Horwitz KB, Mockus MB, Lessey BA (1982) Variant T47D human breast cancer cells with high progesterone-receptor levels despite estrogen and antiestrogen resistance. Cell 28: 633–642 7200400 10.1016/0092-8674(82)90218-5

[bib13] Ibrahim YH, Yee D (2005) Insulin-like growth factor-I and breast cancer therapy. Clin Cancer Res 11: 944s–950s 15701891

[bib14] Jackson JG, White MF, Yee D (1998) Insulin receptor substrate-1 is the predominant signaling molecule activated by insulin-like growth factor-I, insulin, and interleukin-4 in estrogen receptor-positive human breast cancer cells. J Biol Chem 273: 9994–10003 9545345 10.1074/jbc.273.16.9994

[bib15] Jackson JG, Yee D (1999) IRS-1 expression and activation are not sufficient to activate downstream pathways and enable IGF-I growth response in estrogen receptor negative breast cancer cells. Growth Horm IGF Res 9: 280–289 10543935 10.1054/ghir.1999.0113

[bib16] Jackson JG, Zhang X, Yoneda T, Yee D (2001) Regulation of breast cancer cell motility by insulin receptor substrate-2 (IRS-2) in metastatic variants of human breast cancer cell lines. Oncogene 20: 7318–7325 11704861 10.1038/sj.onc.1204920

[bib17] Kastner P, Krust A, Turcotte B, Stropp U, Tora L, Gronemeyer H, Chambon P (1990) Two distinct estrogen-regulated promoters generate transcripts encoding the two functionally different human progesterone receptor forms A and B. EMBO J 9: 1603–1614 2328727 10.1002/j.1460-2075.1990.tb08280.x PMC551856

[bib18] Keydar I, Chen L, Karby S, Weiss FR, Delarea J, Radu M, Chaitcik S, Brenner HJ (1979) Establishment and characterization of a cell line of human breast carcinoma origin. Eur J Cancer 15: 659–670 228940 10.1016/0014-2964(79)90139-7

[bib19] Krishan A (1975) Rapid flow cytofluorometric analysis of mammalian cell cycle by propidium iodide staining. J Cell Biol 66: 188–193 49354 10.1083/jcb.66.1.188 PMC2109516

[bib20] Lavan BE, Fantin VR, Chang ET, Lane WS, Keller SR, Lienhard GE (1997a) A novel 160-kDa phosphotyrosine protein in insulin-treated embryonic kidney cells is a new member of the insulin receptor substrate family. J Biol Chem 272: 21403–21407 9261155 10.1074/jbc.272.34.21403

[bib21] Lavan BE, Lane WS, Lienhard GE (1997b) The 60-kDa phosphotyrosine protein in insulin-treated adipocytes is a new member of the insulin receptor substrate family. J Biol Chem 272: 11439–11443 9111055 10.1074/jbc.272.17.11439

[bib22] Lee AV, Jackson JG, Gooch JL, Hilsenbeck SG, Coronado-Heinsohn E, Osborne CK, Yee D (1999) Enhancement of insulin-like growth factor signaling in human breast cancer: estrogen regulation of insulin receptor substrate-1 expression *in vitro* and *in vivo*. Mol Endocrinol 13: 787–796 10319328 10.1210/mend.13.5.0274

[bib23] Meyer GE, Shelden E, Kim B, Feldman EL (2001) Insulin-like growth factor I stimulates motility in human neuroblastoma cells. Oncogene 20: 7542–7550 11709726 10.1038/sj.onc.1204927

[bib24] Nagle JA, Ma Z, Byrne MA, White MF, Shaw LM (2004) Involvement of insulin receptor substrate 2 in mammary tumor metastasis. Mol Cell Biol 24: 9726–9735 15509777 10.1128/MCB.24.22.9726-9735.2004 PMC525494

[bib25] Oesterreich S, Zhang P, Guler RL, Sun X, Curran EM, Welshons WV, Osborne CK, Lee AV (2001) Re-expression of estrogen receptor alpha in estrogen receptor alpha- negative MCF-7 cells restores both estrogen and insulin-like growth factor-mediated signaling and growth. Cancer Res 61: 5771–5777 11479214

[bib26] Pegram MD, Pietras R, Bajamonde A, Klein P, Fyfe G (2005) Targeted therapy: wave of the future. J Clin Oncol 23: 1776–1781 15755985 10.1200/JCO.2005.11.029

[bib27] Reiss K, Wang JY, Romano G, Tu X, Peruzzi F, Baserga R (2001) Mechanisms of regulation of cell adhesion and motility by insulin receptor substrate-1 in prostate cancer cells. Oncogene 20: 490–500 11313980 10.1038/sj.onc.1204112

[bib28] Richer JK, Jacobsen BM, Manning NG, Abel MG, Wolf DM, Horwitz KB (2002) Differential gene regulation by the two progesterone receptor isoforms in human breast cancer cells. J Biol Chem 277: 5209–5218 11717311 10.1074/jbc.M110090200

[bib29] Rocha RL, Hilsenbeck SG, Jackson JG, VanDenBerg CL, Weng C, Lee AV, Yee D (1997) Insulin-like growth factor binding protein-3 and insulin receptor substrate-1 in breast cancer: correlation with clinical parameters and disease-free survival. Clin Cancer Res 3: 103–109 9815544

[bib30] Sachdev D, Hartell JS, Lee AV, Zhang X, Yee D (2004) A dominant negative type I insulin-like growth factor receptor inhibits metastasis of human cancer cells. J Biol Chem 279: 5017–5024 14615489 10.1074/jbc.M305403200

[bib31] Sachdev D, Li SL, Hartell JS, Fujita-Yamaguchi Y, Miller JS, Yee D (2003) A chimeric humanized single-chain antibody against the type I insulin-like growth factor (IGF) receptor renders breast cancer cells refractory to the mitogenic effects of IGF-I. Cancer Res 63: 627–635 12566306

[bib32] Sachdev D, Yee D (2001) The IGF system and breast cancer. Endocr Relat Cancer 8: 197–209 11566611 10.1677/erc.0.0080197

[bib33] Sartorius CA, Groshong SD, Miller LA, Powell RL, Tung L, Takimoto GS, Horwitz KB (1994) New T47D breast cancer cell lines for the independent study of progesterone B- and A-receptors: only antiprogestin-occupied B-receptors are switched to transcriptional agonists by cAMP. Cancer Res 54: 3868–3877 8033109

[bib34] Sepp-Lorenzino L, Rosen N, Lebwohl DE (1994) Insulin and insulin-like growth factor signaling are defective in the MDA MB-468 human breast cancer cell line. Cell Growth Differ 5: 1077–1083 7848909

[bib35] Shaw LM (2001) Identification of insulin receptor substrate 1 (IRS-1) and IRS-2 as signaling intermediates in the alpha6beta4 integrin-dependent activation of phosphoinositide 3-OH kinase and promotion of invasion. Mol Cell Biol 21: 5082–5093 11438664 10.1128/MCB.21.15.5082-5093.2001 PMC87234

[bib36] Sun XJ, Pons S, Wang LM, Zhang Y, Yenush L, Burks D, Myers Jr MG, Glasheen E, Copeland NG, Jenkins NA, Pierce JH, White MF (1997) The IRS-2 gene on murine chromosome 8 encodes a unique signaling adapter for insulin and cytokine action. Mol Endocrinol 11: 251–262 9013772 10.1210/mend.11.2.9885

[bib37] Surmacz E (2000) Function of the IGF-I receptor in breast cancer. J Mammary Gland Biol Neoplasia 5: 95–105 10791772 10.1023/a:1009523501499

[bib38] Sutherland RL, Lee CS, Feldman RS, Musgrove EA (1992) Regulation of breast cancer cell cycle progression by growth factors, steroids and steroid antagonists. J Steroid Biochem Mol Biol 41: 315–321 1562509 10.1016/0960-0760(92)90357-o

[bib39] Tobe K, Tamemoto H, Yamauchi T, Aizawa S, Yazaki Y, Kadowaki T (1995) Identification of a 190-kDa protein as a novel substrate for the insulin receptor kinase functionally similar to insulin receptor substrate-1. J Biol Chem 270: 5698–5701 7534300 10.1074/jbc.270.11.5698

[bib40] Twentyman PR, Luscombe M (1987) A study of some variables in a tetrazolium dye (MTT) based assay for cell growth and chemosensitivity. Br J Cancer 56: 279–285 3663476 10.1038/bjc.1987.190 PMC2002206

[bib41] Valentinis B, Navarro M, Zanocco-Marani T, Edmonds P, McCormick J, Morrione A, Sacchi A, Romano G, Reiss K, Baserga R (2000) Insulin receptor substrate-1, p70S6K, and cell size in transformation and differentiation of hemopoietic cells. J Biol Chem 275: 25451–25459 10846175 10.1074/jbc.M002271200

[bib42] Vassen L, Wegrzyn W, Klein-Hitpass L (1999) Human insulin receptor substrate-2 (IRS-2) is a primary progesterone response gene. Mol Endocrinol 13: 485–494 10077005 10.1210/mend.13.3.0256

[bib43] von Minckwitz G, Jonat W, Fasching P, du Bois A, Kleeberg U, Luck HJ, Kettner E, Hilfrich J, Eiermann W, Torode J, Schneeweiss A (2005) A multicentre phase II study on gefitinib in taxane- and anthracycline-pretreated metastatic breast cancer. Breast Cancer Res Treat 89: 165–172 15692759 10.1007/s10549-004-1720-2

[bib44] Wang LM, Myers Jr MG, Sun XJ, Aaronson SA, White M, Pierce JH (1993) IRS-1: essential for insulin- and IL-4-stimulated mitogenesis in hematopoietic cells. Science 261: 1591–1594 8372354 10.1126/science.8372354

[bib45] Wen DX, Xu YF, Mais DE, Goldman ME, McDonnell DP (1994) The A and B isoforms of the human progesterone receptor operate through distinct signaling pathways within target cells. Mol Cell Biol 14: 8356–8364 7969170 10.1128/mcb.14.12.8356-8364.1994 PMC359374

[bib46] White MF, Maron R, Kahn CR (1985) Insulin rapidly stimulates tyrosine phosphorylation of a Mr-185 000 protein in intact cells. Nature 318: 183–186 2414672 10.1038/318183a0

[bib47] Zhang X, Kamaraju S, Hakuno F, Kabuta T, Takahashi S, Sachdev D, Yee D (2004) Motility response to insulin-like growth factor-I (IGF-I) in MCF-7 cells is associated with IRS-2 activation and integrin expression. Breast Cancer Res Treat 83: 161–170 14997047 10.1023/b:brea.0000010709.31256.c6

[bib48] Zhang X, Lin M, van Golen KL, Yoshioka K, Itoh K, Yee D (2005) Multiple signaling pathways are activated during insulin-like growth factor-I (IGF-I) stimulated breast cancer cell migration. Breast Cancer Res Treat 93: 159–168 16187236 10.1007/s10549-005-4626-8

[bib49] Zhang X, Yee D (2000) Tyrosine kinase signalling in breast cancer: insulin-like growth factors and their receptors in breast cancer. Breast Cancer Res 2: 170–175 11250706 10.1186/bcr50 PMC138771

[bib50] Zhang X, Yee D (2002) Insulin-like growth factor binding protein-1 (IGFBP-1) inhibits breast cancer cell motility. Cancer Res 62: 4369–4375 12154042

